# Characterization and differentiation of equine experimental local and early systemic inflammation by expression responses of inflammation-related genes in peripheral blood leukocytes

**DOI:** 10.1186/s12917-016-0706-8

**Published:** 2016-06-01

**Authors:** Anne Mette L. Vinther, Peter M. H. Heegaard, Kerstin Skovgaard, Rikke Buhl, Stine M. Andreassen, Pia H. Andersen

**Affiliations:** Department of Large Animal Sciences, Faculty of Health and Medical Sciences, University of Copenhagen, Taastrup, Denmark; Innate Immunology Group, Section for Immunology and Vaccinology, National Veterinary Institute, Technical University of Denmark, Frederiksberg, Denmark; Department of Clinical Sciences, Faculty of Veterinary Medicine and Animal Science, Swedish University of Agricultural Sciences, Uppsala, Sweden

**Keywords:** Cytokines, Endotoxemia, Gene expression, Horses, Inflammatory transition, Innate immunity, Leukocytes, Lipopolysaccharide, Local inflammation, Systemic inflammation

## Abstract

**Background:**

Local inflammation may progress into systemic inflammation. To increase our understanding of the basic immunological processes during transition of equine local inflammation into a systemic state, investigation into the equine systemic immune response to local inflammation is warranted. Therefore, the aim of this study was to investigate the innate peripheral blood leukocyte (PBL) immune response to local inflammation in horses, and to compare this response with the PBL immune response during the early phase of acute systemic inflammation. Expression of 22 selected inflammation-related genes was measured in whole blood leukocytes from 6 horses in an experimental cross-over model of lipopolysaccharide- (LPS-) induced acute synovitis (3 μg LPS intraarticularly; locally inflamed [LI] horses) and endotoxemia (1 μg LPS/kg intravenously; systemically inflamed [SI] horses). Multiple clinical and hematological/biochemical examinations were performed, and serial blood samples were analyzed by reverse transcription quantitative real-time PCR. Post-induction expression profiles of all genes were compared between study groups using principal component analysis (PCA) and hierarchical clustering.

**Results:**

Moderate synovitis and mild systemic inflammation of approximately 24 h duration was confirmed by clinical and paraclinical observations in LI and SI horses, respectively. In the LI group, samples obtained 3–16 h post-injection showed distinct clustering in the PCA compared with baseline levels, indicating a transcriptional response to local inflammation in PBLs in this time interval. There was no clinical or hematological indication of actual systemic inflammation. There was a clear separation of all LI samples from all SI samples in two distinct clusters, indicating that expression profiles in the two study groups were different, independent of time since LPS injection. Co-regulated genes formed four clusters across study groups which were distinctly differently regulated. Only few of individual genes displayed different expression between the study groups at all times after LPS injection.

**Conclusions:**

Local inflammation in horses initiated an innate transcriptional response in PBLs, which differed from the transcriptional response during the early phase of systemic inflammation. This study may provide new insights into the immunobiology of PBLs during the transition of local inflammation into a systemic state.

**Electronic supplementary material:**

The online version of this article (doi:10.1186/s12917-016-0706-8) contains supplementary material, which is available to authorized users.

## Background

Acute inflammation denotes a series of generalized vascular and cellular events that is induced by the innate immune system in response to invading pathogens and tissue trauma [1, 2]. Systemic inflammation arises secondary to local inflammation if pro-inflammatory substances such as pathogen-associated molecular patterns (PAMPs), danger-associated molecular patterns (DAMPs), and perhaps pro-inflammatory cytokines are not successfully contained by local inflammatory processes, and if these pro-inflammatory substances overwhelm the initial systemic host defenses [1, 3, 4]. Activated peripheral blood leukocytes (PBLs), especially neutrophil granulocytes and monocytes, and endothelial cells are regarded key immunological cells in the initiation and progression of systemic inflammatory processes [5, 6].

In the equine clinic, systemic inflammation is a frequent sequela to diseases such as infectious colitis, metritis, peritonitis, pleuropneumonia, and intrauterine/neonatal infections [7]. There is however not always a clear association between type of disease and the development of systemic inflammation; some horses suffering from local inflammation develop systemic inflammation while others apparently do not. The importance of closely monitoring patients at risk of developing systemic inflammation is widely recognized, as rapidly initiated targeted therapy is crucial to the treatment of systemic inflammatory conditions [7, 8]. However, diagnosing early acute systemic inflammation can be a challenge, as signs of disease during the transition of local inflammation into a systemic state inevitably will contain a mix of host responses to both local and systemic inflammatory processes. Also, local inflammation may initiate clinical and paraclinical signs which usually are thought of as evidence of systemic involvement, even though systemic inflammation does not appear to be present. This has been demonstrated in experimental studies of local inflammation in horses in which alterations in body temperature, whole blood white blood cell (WBC) count, and in some cases serum acute phase reactants (APRs), were present in response to LPS-induced synovitis [9–12] and *E. coli*-induced endometritis [13]. A systemic response like this might be due to release of locally produced pro-inflammatory cytokines to circulation, as cytokines are well known initiators of both fever [14], neutrophilia [15, 16], and acute phase responses [17]. Another, more indirect, source of such cytokines might be PBLs and endothelial cells that are activated by pro-inflammatory substances released from locally inflamed tissues. From a clinical point of view, the most specific sign of the early acute stage of systemic inflammation regardless of cause seems to be the presence of pronounced neutropenia [18], indicating that activated neutrophils have marginated in the vasculature or have sequestrated in the perivascular tissue on a generalized level [19, 20].

In human medicine, research into systemic inflammation is focused on complementing clinical and standard paraclinical parameters with immunological markers to optimize future diagnosis and prognosis of systemic inflammatory conditions [21, 22]. In both human and equine research, the traditional way of investigating immune processes during the onset of systemic inflammation is by quantitating inflammatory mediators in serum or their corresponding gene expression in PBLs in response to experimentally induced systemic inflammation [23–27]. However, very little is known about the systemic immune response to local inflammation. To increase our understanding of basic immunological processes during transition of equine local inflammation into a systemic state, investigation of the PBL immune response to local inflammation is warranted. Such investigations might further indicate if specific PBL inflammatory mediators have a diagnostic potential in monitoring equine patients at risk of developing systemic inflammation.

Therefore, the aim of this study was to investigate the innate PBL immune response to local inflammation in horses, and to compare this response with the innate PBL immune response during the early phase of acute systemic inflammation. This was done by measuring the expression of 22 selected inflammation-related genes in whole blood leukocytes in an equine cross-over model of LPS-induced acute synovitis and endotoxemia. This study is part of a larger experiment investigating inflammatory processes during equine LPS-induced acute synovitis and endotoxemia. Clinical results and expression levels of single genes for the endotoxic horses have previously been presented in Vinther et al. [28].

## Methods

### Horses

Six healthy, adult (7.7 ± 5.4 years), Danish Warmblood or Danish Warmblood cross bred horses (2 geldings and 4 non-pregnant mares) with an initial body weight of 490 ± 39 kg were included in the study. The horses were owned by the University of Copenhagen. Prior to the experiment a full clinical examination and hematological and biochemical analyses of blood and radiocarpal synovial fluid were performed, and only horses without clinical or hematological/biochemical signs of acute or chronic inflammation [29] were included in the study. All horses were dewormed with ivermectin (Maximec®) and vaccinated against equine influenza and tetanus (ProteqFlu-Te®) prior to the study. Approximately 2 weeks before initiation of the study, horses were housed at the research station in individual 3 x 4 m box stalls, where they were fed an equine commercial grain mixture twice daily and had access to water and hay ad libitum.

### Experimental design

A cross over study involving experimentally induced endotoxemia and lipopolysaccharide-(LPS) induced synovitis as models for systemic and localized inflammation, respectively, was performed. Horses were randomly assigned to start in one of the two study groups, and the experiments were performed simultaneously on one horse from each study group at a time. All horses had an acclimatization period in the climate controlled experimental facilities (maintained at a temperature of 12 ± 1 °C) of 5 days before initiation of the experiment. To eliminate possible effects of LPS tolerance there was a 4 week wash out period in between treatments [30]. LPS was derived from *Escherichia coli* strain 055:B5 (#L2880, Sigma-Aldrich Denmark). Using a polyurethane jugular catheter (Milacath®, 14G, 150 mm), endotoxemia was induced over one minute with 1 μg/kg LPS diluted in isotonic saline (Baxter A/S, Denmark) to a total volume of 15 mL (systemically inflamed [SI] horses). Synovitis was induced in the left carpal joint by sterile intraarticular injection (Eickinject®, 21G, 40 mm) of 3 μg LPS diluted in 1 mL Ringer’s Acetate (Baxter A/S, Denmark) (locally inflamed [LI] horses). The LPS doses were established on the basis of the biological action of this particular batch of LPS in previous experiments, where the 3 ug per joint dose resulted in a clinically moderate but fully reversible synovitis that lasted for approximately 24 h, and the IV dose of 1 ug/kg resulted in a systemic inflammation with fully reversible clinical signs of inflammation that lasted for approximately 24 h. Due to intraarticular LPS injection failure in one LI horse, this group only consisted of 5 horses.

### Clinical examinations

Examinations comprising general condition, rectal temperature (RT), heart rate (HR), respiratory rate (RR), mucosal membrane color, capillary refill time (CRT), and lameness score (LI horses only) were performed multiple times during the 24-h experimental period for both SI and LI horses (Additional file 1). Lameness was assessed independently by two observers using the AAEP lameness score [31]. Two hours after intraarticular LPS injection the left carpal joint of the LI horses was evaluated for swelling and heat, and synovial fluid total protein and WBC were measured. 2.2 mL of synovial fluid was obtained by aseptic arthrocentesis (EDTA tubes, BD Company), and synovial fluid total protein and WBC was measured by use of a refractometer^1^ and a haemocytometer^2^, respectively. Additional arthrocenteses at post-injection hour (PIH) 4, 8, 16, and 24 were performed as part of a study without relevance for this experiment.

### Blood sampling

Dependent on specific requirements for analyses at each time point for sampling, 7.5–31.5 mL of blood was collected in syringes (Kruuse) through the indwelling jugular venous catheter and immediately transferred to the appropriate blood tubes. The first 5 mL of blood were drawn in a separate syringe and discarded. Baseline samples (PIH 0) were taken immediately before LPS injection. Blood for WBC and total neutrophil granulocyte and lymphocyte counts was collected in EDTA tubes (BD Company) at PIH 0, 1, 2, 3, 4, 6, 8, 12, 16, 20, and 24. Blood for APRs was collected in serum tubes (serum amyloid A (SAA), haptoglobin, iron) or citrate tubes (fibrinogen) (BD Company) at PIH 0, 2, 4, 8, 16, and 24. SAA was measured by immunoturbidometry^3^. Haptoglobin was measured by a biochemical peroxidase assay in duplicate^4^. Iron was measured by colorimetric spectrophotometry^5^. Fibrinogen was measured by the Clauss method in an automated coagulometric analyzer^6^. A standardized volume of 2.5 mL of blood for RNA extraction was collected in PAXgene Blood RNA Tubes (Qiagen/BD Company), which allows for later recovery of total RNA from all cells presents in the blood sample. According to the manufacturer’s instruction PAXgene™ blood tubes were gently inverted 8–10 times after sampling and kept at room temperature for 2 to 24 h, before storage at -80 °C until RNA extraction. Samples from LI horses were collected at PIH 0, 1, 2, 3, 4, 6, 8, 10, 12, 16, 20, and 24. For SI horses, genes of interest were previously found to be maximally differentially expressed within the first 8 h after LPS injection [28]. Thus, only SI samples for PIH 1-8 were included in this study, serving as references for the expression profiles during acute systemic inflammation in these particular horses.

### Target genes

Relative gene expression in PBLs was measured by quantification of specific mRNA for the following 22 genes: *IL1B*, *IL6*, *IL8*, *IL10*, *IL15*, *IL17*, *IL18*, *IL1RN*, *TNF*, *TLR4*, *CD14*, *ITGAM*, *ITGAX*, *SELL, MAPK14*, *CASP3*, *BCL2L1*, *MMP8*, *TIMP1*, *IL6ST*, *CCL5*, and *SOD2* (Additional file 2). As presented in Vinther et al. [28]; these 22 genes were significantly regulated in the SI horses at one or more time points during the first 8 h after intravenous LPS injection. Significant regulation was defined as a statistically significant change in gene expression of at least 2.5 fold compared with baseline levels.

### RT-qPCR

Total RNA extraction and quality analysis, cDNA synthesis, specific primer design, pre-amplification and exonuclease treatment, and qPCR were performed as previously described [28]. Briefly, total cellular RNA was extracted from PAXgene™ blood samples using PAXgene Blood miRNA Kits (Qiagen/BD Company) and treated with RNase-Free DNase sets (Qiagen/BD Company). Concentration and purity of total extracted RNA was determined by spectrophotometric analyses^7^. Samples containing less than 20 ng RNA/μL were evaporated (37 °C) to increase RNA concentration using a SPD111V SpeedVac Concentrator^8^. RNA integrity was estimated using an Agilent 2100 Bioanalyzer^9^ and RNA 6000 Nano Kits (Agilent Technologies). Each total RNA sample was assigned an RNA Integrity Number (RIN) from 1-10, with 10 being non-degraded RNA [32]. Mean RIN value ± SD of all samples was 8.9 ± 0.9.

After extraction RNA was DNase treated a second time and 300 ng of total RNA per sample converted into first-strand cDNA by reverse transcription using a Tprofessional TRIO 3x48^10^ and QuantiTect Reverse Transcription Kits (Qiagen/BD Company) according to the manufacturer’s instructions. For assay validation, two separate cDNA replicates were performed for each RNA sample and a non-reverse transcriptase control was included. The cDNA samples were diluted (1:7.7) in low TE-buffer (VWR-Bie & Berntsen) and stored at -20 °C until pre-amplification procedures.

Gene specific primer pairs were designed as previously described [28] and synthesized at TAG Copenhagen (Denmark) or Sigma-Aldrich (Denmark). Transcript IDs, primer sequences, amplicon lengths, reaction efficiencies, and correlation coefficients are shown in Additional file 2. Primer amplification efficiencies, correlations and dynamic ranges were acquired from standard curves based on 4 separate dilution series (3 representing the whole sample material and 1 representing samples with high levels of *IL6* to specifically meet the dynamic range of this particular gene) of pooled cDNA [28]. Efficiencies between 0.91 and 1.12 and correlations above 0.97 were accepted.

Stocks of 200 nM primer pair mix in low TE-buffer (VWR-Bie & Berntsen, Denmark) were prepared combining equal amounts of all primers used in the study. Primer pair mix, TaqMan PreAmp Master Mix (Applied Biosystems), and cDNA were mixed and incubated at 95 °C for 10 min followed by 16 cycles of 95 °C for 15 s and 60 °C for 4 min. After pre-amplification cDNA was exonuclease treated. An aliquot of the pre-amplified cDNA was saved for preparation of dilution series, and finally cDNA was diluted in low TE-buffer before quantitative real-time PCR (qPCR).

Quantitative PCR was performed in 48.48 Dynamic Array Integrated Fluidic Circuits (IFC) on a BioMark Fluidigm thermocycler^11^ combining 48 samples with 48 primer sets in 2304 separate, simultaneous reactions [33]. Each chip included a non-template control (NTC), a non-reverse transcriptase control, and cDNA dilution pools. Expression data (Cq values in heat maps) and melting curves were acquired using Fluidigm Real-Time PCR Analysis software 3.0.2 (Fluidigm). NTCs and melting curves were used to monitor for non-specific amplification or sample contaminations, and non-reverse transcriptase controls were used to assess potential DNA contamination. For both of these control samples, a minimum of 5 Cq-values between potential signals and a given sample signal were required, and only genes with a single melting peak (within 1 °C) were accepted for further data analyses.

### Pre-processing and quantification of data

Expression data were exported to GenEx5^12^ for pre-processing and quantification. Dilution pools were used as inter plate calibrators. Data was corrected for primer efficiencies for each primer assay individually. Using NormFinder [34] and GeNorm [35] the most stably expressed reference genes (ACTB, TBP, DIMT1, SDHA, HPRT1, and B2M) out of 7 putative candidates were selected for normalization. cDNA technical replicates were averaged. Finally, relative expression levels were established. For each gene of interest the expression level was set to 1 for the sample with the lowest level of expression. Expression levels for the specific gene of interest in all other samples irrespective of horse and time for sampling were then calculated relative to this sample during data transformation from Cq (log2) to relative quantities (relative fold change, linear scale).

### Statistical analyses

#### Clinical and paraclinical data

Clinical and paraclinical data are presented as mean values except for lameness score, which is presented as a range of scores of individual horses. Selected clinical and paraclinical data are depicted graphically using SigmaPlot v. 12.5. Statistical analyses of selected clinical and hematological responses (RT, HR, RR, WBC counts, APRs) were analyzed with a two-way ANOVA random effect (mixed effect) model (R v. 3.1.2) where time and treatment (and their interaction) were fixed effects that influence the mean. A serial Gaussian correlation structure was included to allow for time-dependent correlation within each treatment on each horse and to allow for variation among horses. Distribution of data was evaluated by residual plots and data was log2-transformed to improve the model fits. The t-test was used to compare each time point with the baseline at PIH 0 and to compare time points between treatments groups (APRs only). The Bonferroni correction was used to account for testing multiple disease parameters, and statistical significance was defined as *P* < 0.0063 (significance level 0.05/8 parameters). No correction was made for testing at multiple time points as number of time point varied between parameters.

#### Expression data

All analyses on expression data were performed on log2-transformed, autoscaled data using GenEx5. Data was autoscaled to have similar weights in the analyses regardless of magnitude and variation in expression levels of the 22 genes [36]. To disclose multivariate responses in the study groups, the method of principal component analysis (PCA) was employed, grouping samples or genes based on gene co-expression patterns [37]. In the present study, two PCAs were performed to visualize clusters of individual samples. The first PCA included expression data of all LI samples divided into the following subgroups: PIH 0, PIH 1-2, PIH 3-4, PIH 5-8, PIH 10-16, and PIH 20-24. The second PCA included expression data of SI (PIH 1-8) samples, LI (PIH 3-16) samples, and baseline samples for both study groups.

Based on expression level group means, differences in expression profiles of LI (PIH 3-16) samples in relation to SI (PIH 1-8) samples were further visualized in a heat map combining dendrograms of samples and genes. The dendrograms were performed by using agglomerative clustering, Euclidean distance as the distance measure and Ward’s method as clustering algorithm.

## Results

### Clinical and paraclinical responses to endotoxin

**Fig. 1:**
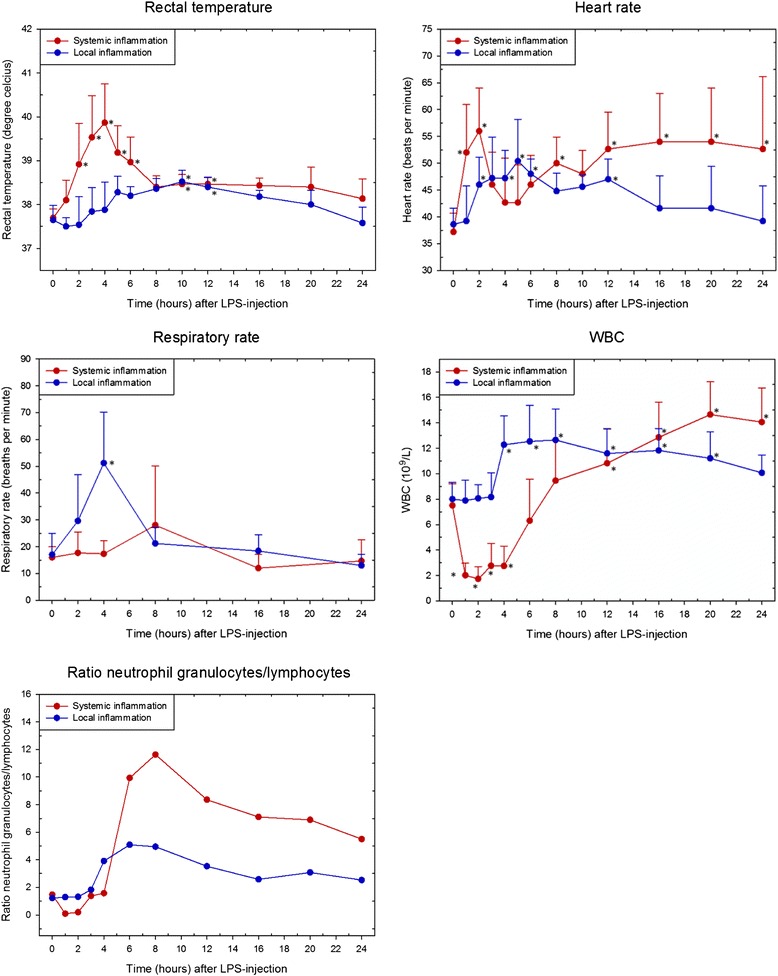
Clinical and hematological responses to LPS. Rectal temperature, heart rate, respiratory rate, white blood cell (WBC) counts, and neutrophil granulocyte/lymphocyte ratio in horses with induced systemic (red) and local (blue) inflammation, respectively. All values except neutrophil granulocyte/lymphocyte ratio are depicted as mean ± SD. Asterisk (*) denotes statistical significant differences compared with baseline levels at PIH 0 (*P* < 0.0063). Please note that clinical and hematological responses for the horses with systemic inflammation have also been presented in the article Vinther et al. [28]. PIH: post-injection hour

**Fig. 2:**
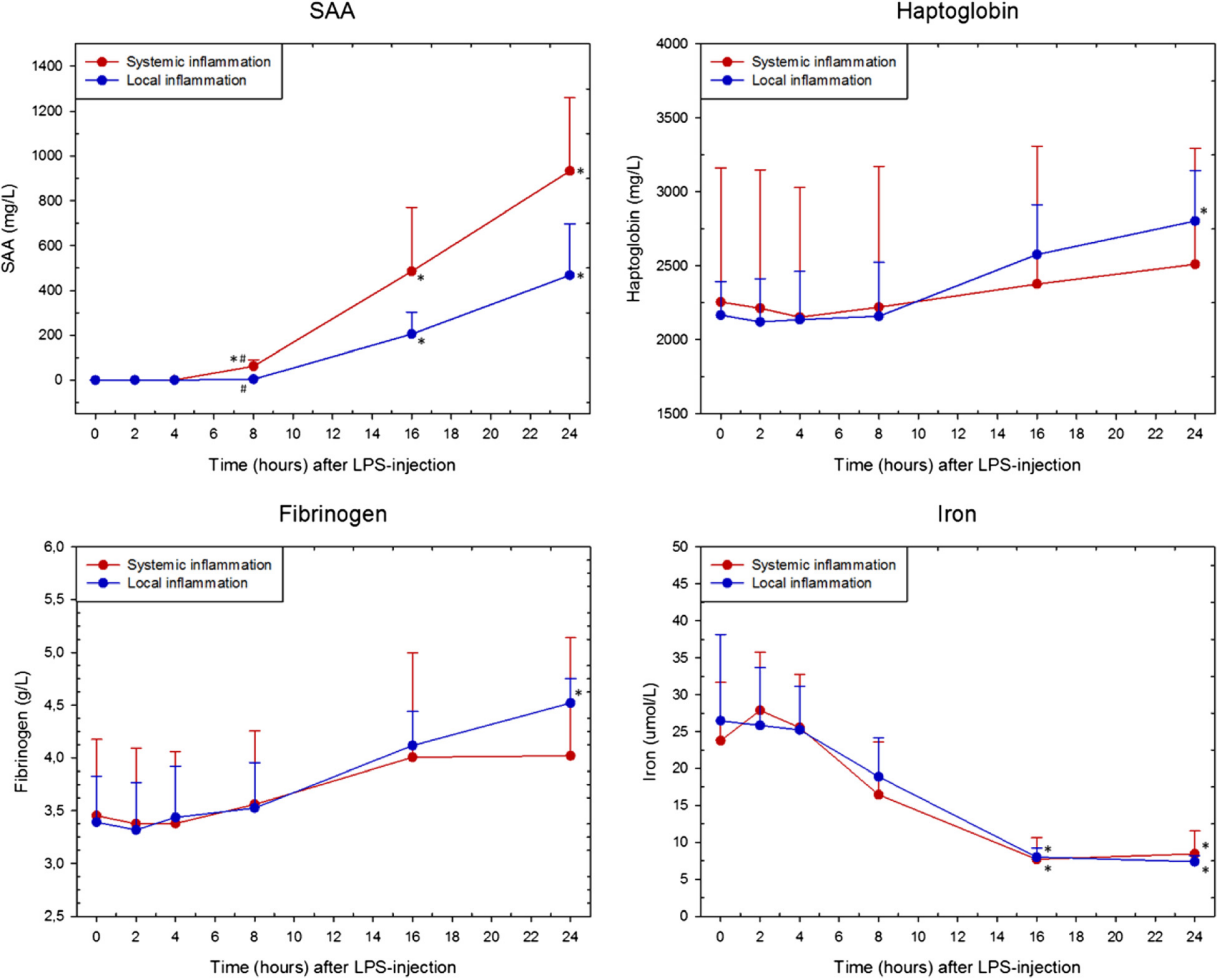
Acute phase responses to LPS. Concentration of serum or plasma serum amyloid A (SAA), haptoglobin, fibrinogen, and iron (mean ± SD) in horses with induced systemic (*red*) and local (*blue*) inflammation, respectively. Asterisk (*) denotes statistical significant differences compared with baseline levels at PIH 0, while hash tag (^#^) denotes statistical significant differences between SI and LI horses (*P* < 0.0063). PIH: post-injection hour

During the acclimatization period all horses were bright, alert, and responsive, and considered healthy on clinical and hematological examinations (data not shown). A graphical presentation of baseline and post-injection results of selected clinical and paraclinical parameters including statistics for both study groups are presented in Figs. 1 and 2. SI horses started to show signs consistent with endotoxemia such as anorexia, mild to moderate colic, fever, tachycardia, and pronounced neutropenic leukopenia within the first two hours post-injection. WBC started to increase after PIH 2 due to an increasing number of circulating neutrophil granulocytes, and at PIH 4 rectal temperature peaked. Within the first 8 h after LPS injection all horses except one had hyperemic, dark red, or cyanotic mucosal membranes and a CRT of 3–4 s. At PIH 8, a mildly elevated rectal temperature and heart rate, an increasing WBC count, decreased appetite, depression, and mild colicky behavior characterized the clinical status. After 24 h horses had an elevated heart rate and WBC count but were all, except for one horse, bright, alert, and responsive. All horses recovered without treatment.

At PIH 2, LI horses were 4 to 5° lame (5° scale), and had mild tachycardia and tachypnea (Fig. 1). There was swelling of the left carpal joint, palpable heat, and elevated synovial fluid total protein (35.2 ± 9.7 g/L) and WBC count (61.2 ± 31.6 x 10^9^ cells/L) (reference values of non-inflamed synovial fluid total protein and WBC approximately 0.2 g/L and 87-167 x 10^6^ cells/L, respectively [38]). During the following 2–6 h horses expressed varying degrees of depression, decreased appetite, mild colic, increased WBC counts, tachycardia, and tachypnea. Rectal temperature was slightly elevated at PIH 5-12. Lameness score was 4 to 5° at PIH 8. At PIH 24, horses were 1 to 3° lame and were bright, alert, and responsive. During the entire disease period all horses had a normal mucosal membrane color and CRT ≤ 2 s. None of the horses received analgesic treatment at any time during the study.

As seen in Fig. 2, serum SAA concentration for SI horses increased from 8 h post-injection until the end of the study whereas for LI horses SAA levels increased at 16 and 24 h of the onset of the study. Iron concentration was decreased after 16 h for both groups. In the LI group, haptoglobin and fibrinogen concentration was increased at PIH 24, while no significant changes were seen for these two acute phase proteins in the SI group. SAA concentration was different between SI and LI horses at PIH 8. Otherwise acute phase proteins did not differ between study groups within the 24-h observational period.

### Expression analyses

**Fig. 3:**
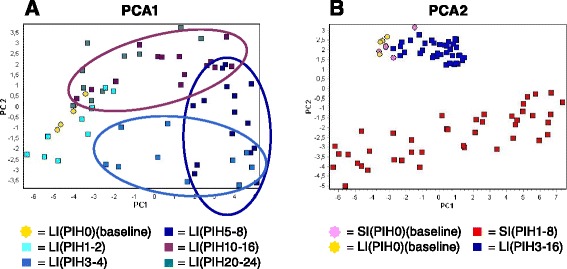
Clustering of samples from systemically and locally inflamed horses. Clustering of samples based on the co-expression patterns of 22 innate genes measured in PBLs. Clustering was performed by use of principal component analysis (PCA) in which individual samples were plotted by their two first principal components. **a** Expression data of all LI samples was divided into the following subgroups: PIH 0 (baselines), PIH 1-2, PIH 3-4, PIH 5-8, PIH 10-16, and PIH 20-24. Ellipses are drawn to highlight the sample subgroups at PIH 3-4, PIH 5-8, and PIH 10-16. **b** Expression data of SI (PIH 1-8) samples, LI (PIH 3-16) samples, and baseline samples for both study groups. PIH: post-injection hour

In the two study groups PCA was used to investigate the innate gene expression response in PBLs based on co-expression patterns of individual samples. Figure 3a and b were obtained by plotting all relevant samples by their two first principal components obtained from all 22 genes. Figure 3a shows distinct clustering of samples from LI (PIH 3-4), LI (PIH 5-8), and to a certain extend LI (PIH 10-16) horses compared with baseline samples. Figure 3b includes all samples from SI horses (PIH 1-8) together with the samples from LI horses in which co-expression patterns in Fig. 3a showed distinct clustering from baseline samples (PIH 3-16). There was a distinct clustering of SI samples from LI samples regardless of time since LPS injections. This clustering was not seen in baseline samples before LPS injection. All samples from SI horses clustered separately from pre-injection samples.

**Fig. 4:**
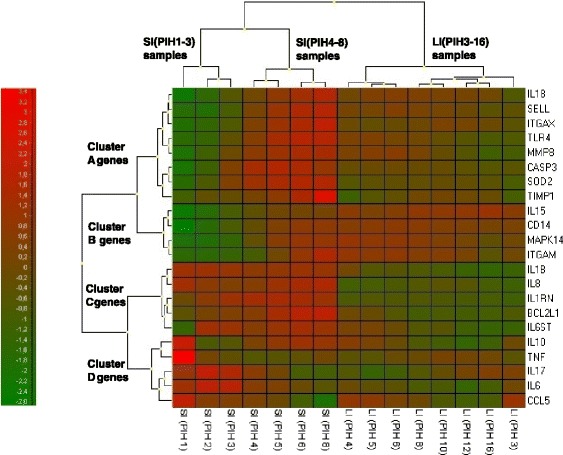
Expression matrix of samples and genes from systemically and locally inflamed horses. Mean expression data for SI (PIH 1-8) samples and LI (PIH 3-16) samples for each of the 22 genes visualized in a heat map. Relative expression levels are visualized by color intensities where red corresponds to high expression and green corresponds to low expression. Please note that baseline samples are not included to indicate baseline levels. The upper dendrogram shows clustering of samples across genes. The dendrogram to the left indicates clustering of genes across samples (time points and study groups). Clustering is based on similarities between samples/genes indicated by the distance at which they are joined. Short distance indicates high similarity. PIH: post-injection hour

A heat map combining two dendrograms was constructed to visualize the expression matrix of samples and genes together to further characterize clustering within samples and genes for SI (PIH 1-8) horses and LI (PIH 3-16) horses based on average expression levels (Fig. 4). As seen in the upper dendrogram, samples grouped in three main clusters: SI (PIH 1-3), SI (PIH 4-8), and LI (PIH 3-16). The response levels of genes, going from green (low expression) to red (high expression) were more pronounced and fluctuating for the SI group. Genes clustered in 4 groups with similar expression patterns (Cluster A-D) across study groups. The genes *IL8* and *IL1RN* and to a lesser degree *IL1B* in Cluster C were transcribed to different levels for SI and LI horses independent of time since injection of LPS. Expression levels of all other genes only differed between SI and LI horses in certain time intervals during the two disease courses. In the SI group, gene expression levels in Cluster A (*IL18* to *TIMP1*) increased from low to high over time, having a markedly different level than those of the LI horses at PIH 1-2 and PIH 5-8, but not at PIH 3-4. Genes in Cluster B (*IL15*, *CD14*, *MAPK14*, *ITGAM*) showed different expression levels between the LI group and the first 3 h post-injection for the SI group. Genes in Cluster D (*IL10*, *TNF*, *IL17*, *IL6*, *CCL5*) were most highly expressed during the first hours in the SI group but otherwise showed heterogeneous transcription patterns.

## Discussion

All horses in the SI and LI group developed classic signs of mild endotoxemia and moderate aseptic synovitis, respectively, and both clinical disease courses were self-limiting and lasted approximately for the expected 24 h. In addition to local inflammation and pain, LI-horses showed the systemic response of fever, increased peripheral blood WBC counts, and alterations in serum acute phase reactants previously described in other studies of equine induced local inflammation [9–11], clinically indicating the systemic presence of pro-inflammatory cytokines [14–17]. A hallmark of acute systemic inflammation is the activation of the endothelium, leading to expression of adhesion molecules and leukocyte margination [5]. This is clinically reflected by a rapidly initiated marked decrease in circulating leukocytes, which could be appreciated in the SI horses as early as one hour after induction of inflammation. In contrast, this hallmark of early systemic inflammation was not detected at any time point in the LI horses (Fig. 1). As all other vital clinical and hematological disease parameters, except for RR, in the LI horses only changed mildly, there is a strong indication that intraarticular LPS injection in these horses had not elicited secondary systemic inflammation.

Figure 3a reveals that samples LI (PIH 3-4) and LI (PIH 5-8) clustered distinctly from baseline samples, while LI (PIH 10-16) samples clustered with a partial overlap to baseline samples. This indicates changes in transcription of innate immune genes in circulating leukocytes approximately 3–16 h after intraarticular LPS injection. To our knowledge, this study is the first in horses to show that LPS-induced local inflammation can initiate a complex transcriptional response of innate immune genes in circulating leukocytes. As the horses by all appearances did not experience actual systemic inflammation, this further implies that the detection of transcription activity of innate inflammatory mediators in circulating leukocytes not necessarily equals a systemic inflammatory process in an individual. Whether the systemic response of fever, increased WBC count, and changes in serum APR concentration was due to the release of pro-inflammatory cytokines from the inflamed joint, or whether it was mediated by cytokines produces by activated PBLs, is unknown. However, the increase in WBC count and rectal temperature was not detectable until after the transcriptional onset of genes in PBLs, indicating a relationship between these signs of disease and activation of PBLs. In contrast to this, there was no PBL transcriptional response in LI horses at PIH 2, where horses were almost non-weight-bearing lame, had mild tachycardia and tachypnea, and had signs of acute joint inflammation such as local swelling and heat and elevated synovial fluid total protein and WBC count. This clinical presentation thus reflected a purely local inflammatory process where tachycardia and tachypnea may have been responses to pain from the inflamed joint [39]. Our results thus suggest that elevated body temperature and an increased WBC count may be more indicative of the presence of innate inflammatory mediators in circulation than tachycardia and tachypnea. However, WBC was still significantly elevated at PIH 20 where the systemic expression response had abated.

The presence of inflammatory mediators in circulation in response to local inflammation has only been sparsely investigated in horses. In Cook et al. [40], increased serum levels of the lipid mediators prostaglandin E_2_ and thromboxane B_2_ were found after ischemia-induced local inflammation of the jejunum, and in a study by Campebell et al. [11], no difference in serum TNF-*a* concentration could be detected in LPS-induced synovitis despite significant changes in body temperature and circulating white blood cell composition in response to LPS. In induced endometritis in mares, transcription of the cytokines *IL1B*, *IL6*, *IL8*, *IL10*, *TNF*, and *IL1RN* were investigated in serial samples after inflammatory induction, but no differences were found when compared with pre-induction levels [41]. In dogs, mild changes in WBC counts and in markers of coagulation (activated partial thromboplastin time, fibrinogen, protein-C, and -S) were seen in peripheral blood in response to experimentally induced localized soft tissue inflammation [42]. Increased levels of for example serum IL-6, IL-10, and HMGB-1 were reported in pigs and mice in more severe models of local inflammation such as multiple trauma [43, 44] and acute kidney injury [45]. Even though the exact mechanisms determining whether local inflammation is strictly contained, mediates a systemic response, or initiates actual systemic inflammation are unknown, the severity of the initial insult is probably an important factor. A general dose dependency of the *in vivo* response to LPS has previously been shown in an experimental study in cattle [46]. Furthermore, a study on equine LPS-induced synovitis by Palmer and Bertone [10] specifically supports a dose dependency in the transition of local inflammation to a systemic state, as signs of systemic involvement (fever, altered leukocyte differential count) were only observed after high intraarticular injection doses of LPS. Factors such as pathogen virulence [47, 48] and immune competence of the host [49, 50] most likely contribute to determine the outcome of local inflammation.

In the present study, the PBL transcriptional response to local inflammation at PIH 3-16 was compared with the PBL transcriptional response during the early phase of acute systemic inflammation. The clear separation of all LI samples from all SI samples (Fig. 3b) indicates that expression profiles in the two study groups were substantially different independent of time since LPS injection. In this study, expression profiles of innate immune genes in circulating leukocytes could thus differentiate horses with systemic inflammation from horses with local inflammation and a secondary systemic transcriptional response, during both onset and progression of inflammation. For comparison, pronounced leukopenia, which is pathognomonic for systemic inflammation, was only seen in SI horses for approximately 4 h (Fig. 1). Like WBC, the APRs SAA, haptoglobin, fibrinogen, and iron are also widely used in the diagnosis of inflammation in both veterinary and human medicine [17, 51–53]. While transcription of leukocyte genes in the present study could be detected within the first few hours after LPS injection in both study groups, the acute phase reactants did not significantly change until PIH 8 at the earliest. A delay in acute phase responses compared with cytokine responses is not surprising as cytokines are well known mediators of APRs [17]. It was however remarkable that the 24-h acute phase responses were similar between SI and LI horses despite the two distinctly different gene expression profiles.

To further characterize differences in the PBL transcriptional responses in the two study groups, a heat map combining two dendrograms was constructed using mean expression values for horses for SI (PIH 1-8) and LI (PIH 3-16) samples (Fig. 4). Color intensities in the heat map suggest that the expression response for the SI group in general was more pronounced and with greater fluctuations than for the LI group. This may also be appreciated by the scattered cluster of individual SI samples and the much tighter cluster of individual LI samples in Fig. 3b. The clustering of study groups seen in Fig. 3b is supported by the upper dendrogram in Fig. 4, which furthermore suggests subgrouping of SI samples in PIH 1-3 and PIH 4-8. The subgrouping of SI samples indicates a marked change in the expression profile for SI horses as the disease course progressed, which was likely due to the transition from relative lymphocytosis to relative neutrophilia. The dependence of gene expression results upon the composition of the various types of white blood cells is supported by both equine [54] and human [55, 56] studies and is for the present experiment discussed in detail in Vinther et al. [28]. The heat map suggests that especially IL6, IL17, and most of the genes in Cluster A contributed to subgrouping of the inflammatory response in the SI group.

The 22 genes included in the study clustered in four main groups based on their temporal expression patterns (Cluster A-D, Fig. 4). Grouping of the genes *IL8*, *IL1RN*, and *IL1B* (Cluster C) was also seen in a study by Prabhakar et al*.* [57], in which 16 different genes were clustered by similarities in time profiles during a 24-h course of human LPS-induced systemic inflammation. In the present study, genes within especially Cluster A and B tended to follow the same expression pattern in the SI group, which indicates that the panel of genes could probably be reduced within these clusters without affecting the overall clustering analyses. Assessed visually, only few of the specific genes displayed distinctly different expression levels between study groups, irrespective of time since LPS injection. Except for *IL8*, *IL1RN*, and *IL1B* which seemed to be consistently higher expressed in SI-horses, single gene expressions thus only discriminated LI samples from SI samples in certain time intervals. *IL1RN* has not previously been investigated in systemic inflammation in the horse, and while *IL8* and *IL1B* seem to be differentially expressed in equine experimental systemic inflammation [25, 27, 58], the transcriptional response of these genes in equine septic patients is more unclear [54, 59–61].

By evaluating expression profiles across the 22 genes instead of expression levels of each gene one by one, a more detailed and comprehensive picture of the complex immunological states of the cells was obtained. Multivariate methods for analysis such as PCA and hierarchical clustering are not frequently used in studies of innate immune gene expressions in large animals even though the well described interactions of inflammatory mediators [23, 62] make it a biologically relevant approach. Theoretically, the optimal experimental model to study innate immune processes during the transition of local inflammation into a systemic state would be a model in which systemic inflammation was obtained by inducing local inflammation. To our knowledge, no such experimental model is however well characterized in the horse and we therefore used the traditional intra-articular LPS model as a model for a localized inflammation, even though this may not be the most frequent source of a systemic inflammation. Another limitation of the study includes the fact that the group of horses was quite different according to gender, age, and origin. Furthermore, the complete clinical history of the horses prior to the inclusion examinations was not known, leaving questions on possible endotoxin tolerance due to previous endotoxin related diseases. However, none of the experimental horses showed a decreased pyrogenic response or lack of illness behavior, which are considered clinical hallmarks of tolerance [30, 63]. Additionally, induced in vivo endotoxin tolerance in horses subsides within 2-3 weeks [30], meaning that the risk of endotoxin tolerance in the experimental horses was low. The 4 week wash out period between the two challenges reduced the risk of induction of endotoxin tolerance, and the cross over study minimized effects of the large individual variation known to characterize the innate immune response [55, 64, 65]. The clear clustering of genes described here therefore represent robust findings, despite the low number of experimental horses and their different backgrounds. In the present study, by restricting samples in the SI group to the first 8 h after LPS injection, this model of systemic inflammation was representative of the expression profiles during the onset and progression of early systemic inflammation. Since there is very little knowledge of the possible PBL response to local inflammation, samples from LI horses were included covering the complete course of disease from clinical onset to approximate recovery (PIH 1-24).

To our knowledge, this is the first study in horses that specifically assesses the PBL transcriptional response to local inflammation and describes it in the context of onset and early development of systemic inflammation. New insights into the molecular mechanisms involved in the transition of local inflammation into a systemic state is needed for future advances in the diagnostic opportunities of early systemic inflammation in horses. A recent study by Hooijberg et al. [66] highlights the need for this to supplement standard clinical and paraclinical parameters in the differentiation of equine local and systemic inflammation. The study retrospectively investigated the diagnostic efficacy and combined predictive capability of the myeloperoxidase index (MPXI), and plasma fibrinogen, iron, and SAA concentrations for the diagnosis of systemic inflammation, local inflammation, or non-inflammatory disease in hospitalized horses. Based on quantitative results of these markers, diagnostic guidelines could not be formulated by Hooijberg and colleagues. The group of systemically inflamed horses in the study did not specifically represent early systemic inflammation, but given the results of the APRs in the present study this might not have changed the conclusion. In the present study, an important finding with direct clinical implication is the fact that self-limiting, moderate, local inflammation of brief duration mediated transcription of inflammation-related genes in PBLs. This suggests that a substantial part of equine patients with local inflammatory conditions are exposed to inflammatory mediators on a systemic level, emphasizing the relevance of close monitoring of this patient group.

## Conclusions

The results in this study showed that LPS-induced local inflammation in horses could initiate a transcriptional response of inflammation-related genes in circulating leukocytes, and that such a response not necessarily equals a systemic inflammatory process in the individual. The co-expression patterns of multiple genes differentiated horses with a systemic transcriptional response to local inflammation from horses with early systemic inflammation, independent of the progression of inflammatory states. Only few of the individual genes displayed distinctly different expression levels between study groups irrespective of time since LPS injection. This study may provide new insights into the immunobiology of PBLs during the transition of local inflammation into a systemic state and encourage further research in the subject.

## Abbreviations

APR, acute phase reactant; CRT, capillary refill time; DAMP, damage associated molecular patterns; HR, heart rate; LI, locally inflamed; LPS, lipopolysaccharide; PAMP, pathogen-associated molecular pattern; PCA, principal component analysis; PIH, post-induction hour; RIN, RNA integrity number; RR, respiratory rate; RT, rectal temperature; RT-qPCR, reverse transcription quantitative real-time polymerase chain reaction; SAA, serum amyloid A; SI, systemically inflamed; WBC, white blood cell.

## Additional files

Additional file 1: Time-table of clinical examinations performed during the 24-h experimental period. All procedures were carried out in a similar manner for both systemically and locally injected (LI) horses, except only LI horses underwent lameness evaluation. PIH: post-injection hour. (DOCX 14 kb) Additional file 2: Primers and reaction conditions. *PCR efficiency and correlation coefficient for IL6 is calculated based on a specific high-responder standard curve made from dilution series of a pool of cDNA samples showing high expression levels of the implicated gene. *r* ^2^ = correlation coefficient. (DOCX 28 kb)

## Footnotes

1 Clinical Refractometer T2, Atago.

2 Marienfeld, Th. Geyer.

3 LZ test SAA, EIKEN Chemocal Co., Tokyo, Japan.

4 Phase Range Hp Assay, Tridelta Development Ltd., Kildare, Ireland.

5 ADVIA 1650, Siemens, Lyngby, Denmark.

6 ACL 9000, Instrumentation Laboratory, Barcelona, Spain.

7 NanoDrop ND-1000 spectrophotometer, Saveen and Werner AB, Limhamn, Sweden.

8 SPD111V SpeedVac Concentrator, Thermo Scientific, Slangerup, Denmark.

9 Agilent 2100 Bioanalyzer, Agilent Technologies, Naerum, Denmark.

10 Tprofessional TRIO 3x48, Fisher Scientific Denmark, Slangerup, Denmark

11 48.48 Dynamic Array Integrated Fluidic Circuits and BioMark Fluidigm thermocycler, Fluidigm, California, USA.

12 GenEx5, MultiD, Göteborg, Sweden.
