# Supplementary material for: Characterization and differentiation of equine experimental local and early systemic inflammation by expression responses of inflammation-related genes in peripheral blood leukocytes
Source: BMC Vet Res. 2016 Jun 1;12:83. doi: 10.1186/s12917-016-0706-8 (PMC4888743; doi:10.1186/s12917-016-0706-8)
Supplement: Additional file 1: — Time-table of clinical examinations performed during the 24-h experimental period. All procedures were carried out in a similar manner for both systemically and locally injected (LI) horses, except only LI horses underwent lameness evaluation. PIH: post-injection hour. (DOCX 14 kb) [file 12917_2016_706_MOESM1_ESM.docx]

| Para- PIH  meter | 0 | 1 | 2 | 3 | 4 | 5 | 6 | 8 | 10 | 12 | 16 | 20 | 24 |
| --- | --- | --- | --- | --- | --- | --- | --- | --- | --- | --- | --- | --- | --- |
| General condition | x | x | x | x | x | x | x | x | x | x | x | x | x |
| Rectal temperature | x | x | x | x | x | x | x | x | x | x | x | x | x |
| Heart rate | x | x | x | x | x | x | x | x | x | x | x | x | x |
| Respiratory rate | x |  | x |  | x |  |  | x |  |  | x |  | x |
| Mucosal membrane color | x | x | x | x | x | x | x | x | x | x | x | x | x |
| Capillary refill time | x | x | x | x | x | x | x | x | x | x | x | x | x |
| Lameness score (LI horses only) | x |  | x |  | x |  |  | x |  |  | x |  | x |
