# Supplementary material for: Characterization and differentiation of equine experimental local and early systemic inflammation by expression responses of inflammation-related genes in peripheral blood leukocytes
Source: BMC Vet Res. 2016 Jun 1;12:83. doi: 10.1186/s12917-016-0706-8 (PMC4888743; doi:10.1186/s12917-016-0706-8)
Supplement: Additional file 2: — Primers and reaction conditions. *PCR efficiency and correlation coefficient for IL6 is calculated based on a specific high-responder standard curve made from dilution series of a pool of cDNA samples showing high expression levels of the implicated gene. r 2 = correlation coefficient. (DOCX 28 kb) [file 12917_2016_706_MOESM2_ESM.docx]

| **Gene functional class** | **Gene**  **symbol** | **Gene name** | **Transcript ID** | **Sequence 5´-3´** | **Amplicon length (bp)** | **PCR efficiency** | **r^2^** |
| --- | --- | --- | --- | --- | --- | --- | --- |
| Interleukins | IL1B | Interleukin 1-beta | ENSECAT00000000066 | F: CCTACAGCTGGAGACAGTAGACC  R: TGGGGTACATTGCAGACTCA | 119 | 1.03 | 0.99 |
|  | IL6* | Interleukin 6 | [ENSECAT00000017492](http://www.ensembl.org/Equus_caballus/Transcript/Exons?db=core;g=ENSECAG00000016482;r=4:54422007-54426062;t=ENSECAT00000017492) | F: GCAAAAGATGAAGAATCCAGAAG  R: TCTGTGAATGCAGCTTAGCC | 80 | 0.90 | 0.98 |
|  | IL8 | Interleukin 8 | ENSECAT00000016212 | F: CGTTTTTGAAGAGAGCTGAGG  R: GCTTGAAGTTTCATTGGCATC | 114 | 1.05 | 0.99 |
|  | IL10 | Interleukin 10 | ENSECAT00000009338 | F: GCCTTCAGTAAGCTCCAAGAG  R: CCCTAGGATGCTTCAGTTTTTC | 117 | 1.08 | 0.97 |
|  | IL15 | Interleukin 15 | ENSECAT00000015025 | F: GAGGCTGGCATTCATGTCTT  R: CTGCCAGTTTGCCTCTGTTT | 72 | 1.02 | 0.99 |
|  | IL17 | Interleukin 17 | [ENSECAT00000004849](http://www.ensembl.org/Equus_caballus/Transcript/Exons?db=core;g=ENSECAG00000004258;r=16:34013403-34027100;t=ENSECAT00000004849) | F: GGAGGCATGAAAGGATCAAG  R: TCAGATGGGTAAACCACAAGAA | 85 | 1.08 | 0.97 |
|  | IL18 | Interleukin 18 | [ENSECAT00000016149](http://www.ensembl.org/id/ENSECAT00000016149) | F: TGGCAGGCTTGAACCTAAAC  R:CACAGGTTGATTTCCCTGGT | 82 | 1.03 | 1.00 |
|  | IL1RN | Interleukin 1 receptor antagonist | ENSECAT00000005750 | F: ACAAATGTGGCTCCTCCAAG  R: TTTCAGAGCGTCAGAAGTGC | 111 | 1.03 | 1.00 |
| TNF super family | TNF | Tumor necrosis factor | ENSECAT00000002048 | F: GTTGTAGCAAACCCCCAAG  R: GGTTGTCTGTCAGCTTCACG | 94 | 1.11 | 0.99 |
| Pattern recognition receptors | TLR4 | Toll-like receptor 4 | [ENSECAT00000010707](http://www.ensembl.org/Equus_caballus/Transcript/Summary?db=core;g=ENSECAG00000010339;r=25:21966163-21976507;t=ENSECAT00000010707) | F: GCCAGGGAAAGTCAACTCAA  R: TGGGAGACGATGTCCTTTTC | 94 | 1.03 | 0.99 |
|  | CD14 | CD14 | [ENSECAT00000001658](http://www.ensembl.org/Equus_caballus/Transcript/Exons?db=core;g=ENSECAG00000001784;r=14:36269120-36270284;t=ENSECAT00000001658) | F: CTGCCCGTGGTGAGTAATCT  R: GGAGTTCTGGTCTTGCTGCT | 81 | 1.04 | 1.00 |
| Adhesion molecules | ITGAM | CD11b, integrin alpha M | [ENSECAT00000019583](http://www.ensembl.org/Equus_caballus/Transcript/Exons?db=core;g=ENSECAG00000017418;r=13:18554880-18591972;t=ENSECAT00000019583) | F: GTGAGGGCTCAGACGGATAC  R: CCAGTGATGAGAGCCAGGAG | 110 | 1.01 | 0.99 |
|  | ITGAX | CD11c, integrin alpha X | [ENSECAT00000026050](http://www.ensembl.org/Equus_caballus/Transcript/Summary?db=core;g=ENSECAG00000024082;r=13:18521555-18539975;t=ENSECAT00000026050) | F: AGAGCCCAGATGGAGATGG  R: CACAGAGCTGCCCACAATAA | 75 | 1.02 | 1.00 |
|  | SELL | Selectin L | [ENSECAT00000021448](http://www.ensembl.org/Equus_caballus/Transcript/Summary?db=core;g=ENSECAG00000020122;r=5:6105827-6122462;t=ENSECAT00000021448) | F: CAACCTGTCAAGTGATTCAGTG  R: AGCAGTTGAAGGTGCATGTG | 114 | 0.99 | 0.99 |
| Kinases | MAPK14 | Mitogen-activated protein kinase 14, P38 mitogen-activated protein kinase | [ENSECAT00000015606](http://www.ensembl.org/Equus_caballus/Transcript/Exons?db=core;g=ENSECAG00000014175;r=20:36052316-36094294;t=ENSECAT00000015606) | F: GATGAATGGAAAAGCCTGACC  R: AGTGAAGCGGGATCAAGAGA | 116 | 1.06 | 0.99 |
| Apoptosis-related molecules | CASP3 | Caspase 3 | [ENSECAT00000023864](http://www.ensembl.org/Equus_caballus/Transcript/Exons?db=core;g=ENSECAG00000022197;r=27:25405960-25413902;t=ENSECAT00000023864) | F: CACAGCACCTGGTTACTATTCC  R: GCGTACAGCTTCAGCATAGC | 87 | 1.05 | 1.00 |
|  | BCL2L1 | BCL2-like 1, BCL-XL | [ENSECAT00000018124](http://www.ensembl.org/Equus_caballus/Transcript/Exons?db=core;g=ENSECAG00000017223;r=22:22703756-22746101;t=ENSECAT00000018124) | F: GGCTGGGACACCTTTGTG  R: AGAACCACACCAGCCACAGT | 116 | 1.07 | 0.99 |
| Enzymes | MMP8 | Matrix metallopeptidase 8 | [ENSECAT00000022856](http://www.ensembl.org/Equus_caballus/Transcript/Exons?db=core;g=ENSECAG00000021476;r=7:12616281-12626258;t=ENSECAT00000022856) | F: CTGGAGATATGATAACCAAAGACG  R: CTTGCTGGAAAACTGCATCA | 107 | 1.11 | 1.00 |
|  | TIMP1 | Metallopeptidase inhibitor 1 | [ENSECAT00000014937](http://www.ensembl.org/Equus_caballus/Transcript/Exons?db=core;g=ENSECAG00000014259;r=X:38635460-38637390;t=ENSECAT00000014937) | F: GGGGAATGCTCAGTGTTTC  R: TCTGGAAGCCCTTGTCAGAG | 110 | 1.03 | 0.99 |
| Cytokine receptors | IL6ST | Interleukin 6 signal transducer, glycoprotein 130 | [ENSECAT00000021352](http://www.ensembl.org/Equus_caballus/Transcript/Exons?db=core;g=ENSECAG00000019750;r=21:16399502-16454806;t=ENSECAT00000021352) | F: GCCCAGACAACAGTATTTCAAAC  R: CTGATGAACCTTGCTTTGACC | 92 | 1.03 | 0.99 |
| Chemokines | CCL5 | Chemokine (C-C motif) ligand 5 | [ENSECAT00000026840](http://www.ensembl.org/Equus_caballus/Transcript/Exons?db=core;g=ENSECAG00000024888;r=11:37339220-37345300;t=ENSECAT00000026840) | F: CCAGCAGTCGTCTTTGTCAC  R: GCCCTCCAATCCTAGCTCAT | 110 | 1.06 | 0.99 |
| Reactive oxygen species | SOD2 | Superoxid dismutase 2 | [ENSECAT00000012435](http://www.ensembl.org/Equus_caballus/Transcript/Exons?db=core;g=ENSECAG00000011797;r=31:2049322-2057348;t=ENSECAT00000012435) | F: CTGCAGGGAACAACAGGTC  R: CACATTCCAAATGGCTTTCA | 114 | 1.01 | 0.99 |
| Housekeeping genes | TBP | TATA box binding protein | [ENSECAT00000008864](http://www.ensembl.org/Equus_caballus/Transcript/Exons?db=core;g=ENSECAG00000008516;r=31:10270757-10291470;t=ENSECAT00000008864) | F: CACCAGCAGTTTAGTAGTTATGAGC  R: AGGAGAACAATTCTGGGTTTGA | 80 | 1.10 | 0.99 |
|  | DIMT1 | Dimethyladenosine transferase 1 homolog | ENSECAT00000014730 | F: GACTTCATCAGATTGCTACATGG  R: TTCCAGTTTCTTGACTTGAGTTTG | 100 | 1.04 | 1.00 |
|  | SDHA | Succinate dehydrogenase complex, subunit A | ENSECAT00000003106 | F: AAGACCGGGAAGGTCTCG  R: TCCGTTCCCATCAGTAGGAG | 109 | 1.01 | 1.00 |
|  | ACTB | Actin-beta | ENSECAT00000016856 | F: CAGTGGCATCCACGAAACTA  R: AGCACTGTGTTGGCGTACAG | 84 | 1.02 | 0.99 |
|  | HPRT1 | Hypoxanthine phosphoribosyltransferase 1 | [ENSECAT00000018496](http://www.ensembl.org/Equus_caballus/Transcript/Summary?db=core;g=ENSECAG00000017534;r=X:106897692-106921402;t=ENSECAT00000018496) | F: CAGGACTGAACGGCTTGC  R: CCAGCAGGTCAGCAAAGAAT | 107 | 1.01 | 0.99 |
|  | B2M | Beta-2-microglobulin | [ENSECAT00000001009](http://www.ensembl.org/Equus_caballus/Transcript/Summary?db=core;g=ENSECAG00000000685;r=1:144492381-144497809;t=ENSECAT00000001009) | F: TTACTCACGTCACCCAGCAG  R: ATTTCAATCTCAGGCGGATG | 84 | 1.12 | 0.974 |
|  | GAPDH | Glyceraldehyd-3-phosphate dehydrogenase | ENSECAT00000023721 | F: CAAGCTCATTTCCTGGTATGAC  R: TTACTCCTTGGAGGCCATGT | 85 | 1.09 | 0.994 |
